# RecurIndex-derived molecular features associated with total pathological complete response in triple-negative breast cancer: An exploratory prospective cohort study with single-cell transcriptomic contextualization

**DOI:** 10.1080/15384047.2026.2743407

**Published:** 2026-10-10

**Authors:** Meilin Chen, Xiwen Yan, Qifan Chen, Jiayi Bei, Zheng Pan, Tian Zhang, Weihao Cheng, Lu Xu, Feng Jin, Yan Gao, Zining Jin, Ang Zheng

**Affiliations:** a Beijing Chaoyang Hospital, Capital Medical University, Beijing Chaoyang Hospital, Beijing, China; b Department of Breast Surgery, The First Hospital of China Medical University, Shenyang, China; c Department of Medical Oncology, The First Hospital of China Medical University, Shenyang, China; d Department of Human Anatomy, School of Basic Medical Sciences, Capital Medical University, Beijing, China

**Keywords:** Triple-negative breast cancer, neoadjuvant chemotherapy, pathological complete response, RecurIndex, gene expression signature

## Abstract

**Background:**

Reliable biomarkers for predicting neoadjuvant chemotherapy (NAC) response in triple-negative breast cancer (TNBC) remain limited. We explored whether RecurIndex-derived molecular features were associated with total pathological complete response (tpCR).

**Methods:**

Seventy-four patients with TNBC treated with dose-dense NAC were analyzed. Associations between RecurIndex risk scores, 18 core genes, and tpCR were assessed using logistic regression and ROC analyses. Clinical–molecular models were internally validated by bootstrap resampling. Public single-cell RNA-seq data were used for biological contextualization.

**Results:**

Twenty-nine patients (39.2%) achieved tpCR. The distant recurrence score, was significantly associated with tpCR. OBSL1, BLM, and CCR1 were identified as candidate tpCR-associated genes. The model combining OBSL1, BLM, and clinical nodal stage achieved an AUC of 0.838 and an optimism-corrected AUC of 0.799.

**Conclusions:**

RecurIndex-derived molecular features were associated with tpCR in TNBC. These exploratory findings warrant validation in independent cohorts.

## Introduction

Triple-negative breast cancer (TNBC), characterized by the absence of estrogen receptor (ER), progesterone receptor (PR), and human epidermal growth factor receptor 2 (HER2), accounts for approximately 15%–20% of breast cancers and is associated with aggressive clinical behavior and substantial molecular heterogeneity.^1^ ^,^ ^2^ Neoadjuvant chemotherapy (NAC) remains a key treatment strategy for patients with stage II–III TNBC, and achievement of pathological complete response (pCR) is strongly associated with favorable long-term outcomes.^3-5^ However, response to NAC varies considerably among patients, and currently available clinicopathological factors, including tumor stage and Ki-67, provide only limited ability to predict treatment response.^6^ ^,^ ^7^ Therefore, reliable molecular biomarkers capable of identifying patients with different probabilities of achieving pCR remain clinically needed.

RecurIndex is a 28-gene RT-qPCR–based expression assay developed and clinically evaluated primarily in Asian breast cancer populations. The assay was originally designed to estimate local and distant recurrence risk and to assist postoperative treatment decision-making, particularly in hormone receptor–positive, HER2-negative breast cancer.^8-10^ The panel contains 18 functional target genes together with 10 reference genes and captures biological processes related to cell proliferation, cell-cycle regulation, DNA damage repair, and immune response.^11^ Importantly, several of these biological processes are also closely involved in chemotherapy sensitivity and resistance in TNBC.^8^ ^,^ ^12^ ^,^ ^13^ We therefore hypothesized that the RecurIndex-derived risk scores and individual molecular features might provide information regarding NAC response in this setting. However, the potential value of RecurIndex in the neoadjuvant setting of TNBC has not been systematically investigated.

To address this question, we conducted a multicenter, prospective, exploratory observational cohort study in patients with TNBC receiving dose-dense or dose-intensified NAC. Using pretreatment core biopsy specimens, we first evaluated the associations of the RecurIndex-derived local recurrence risk (LRR) and distant recurrence risk (DR) scores with total pathological complete response (tpCR). We subsequently investigated the individual genes within the panel to identify molecular features associated with tpCR and developed clinical–molecular prediction models. Finally, publicly available single-cell RNA-sequencing data were used to provide biological context for the candidate genes identified in the clinical cohort.

## Methods

### Study design and study population

This multicenter, prospective observational cohort study enrolled patients with TNBC from The First Affiliated Hospital of China Medical University, The Fourth Affiliated Hospital of China Medical University, and Liaoning Cancer Hospital, with The First Affiliated Hospital of China Medical University serving as the coordinating center. All participating centers followed a unified protocol for pretreatment biopsy, molecular testing, NAC, surgery, and pathological assessment.

The study was initially planned to enroll approximately 100 patients. However, because the number of patients meeting all eligibility requirements during the predefined study period was lower than anticipated, the prespecified enrollment target was not reached. A total of 78 eligible patients were consecutively enrolled, of whom four were excluded because of incomplete NAC, failure of biopsy sample quality control, or absence of surgery and postoperative pathological evaluation, leaving 74 patients for the final analysis (Figure 1).

No formal sample size calculation based on a prespecified effect size was performed because this study was designed as an exploratory prospective cohort. Previous studies reported pCR rates of approximately 30%–40% in patients with TNBC receiving NAC. In the present cohort, 29 of 74 patients (39.2%) achieved tpCR, consistent with these previously reported response rates.^14^ ^,^ ^15^ Although the planned enrollment target was not reached, the observed number of tpCR events allowed exploratory evaluation of associations between RecurIndex-derived features and treatment response. Given the limited number of outcome events, multivariable model complexity was deliberately restricted, and bootstrap resampling was used for internal validation and assessment of model optimism.

**Figure 1.**
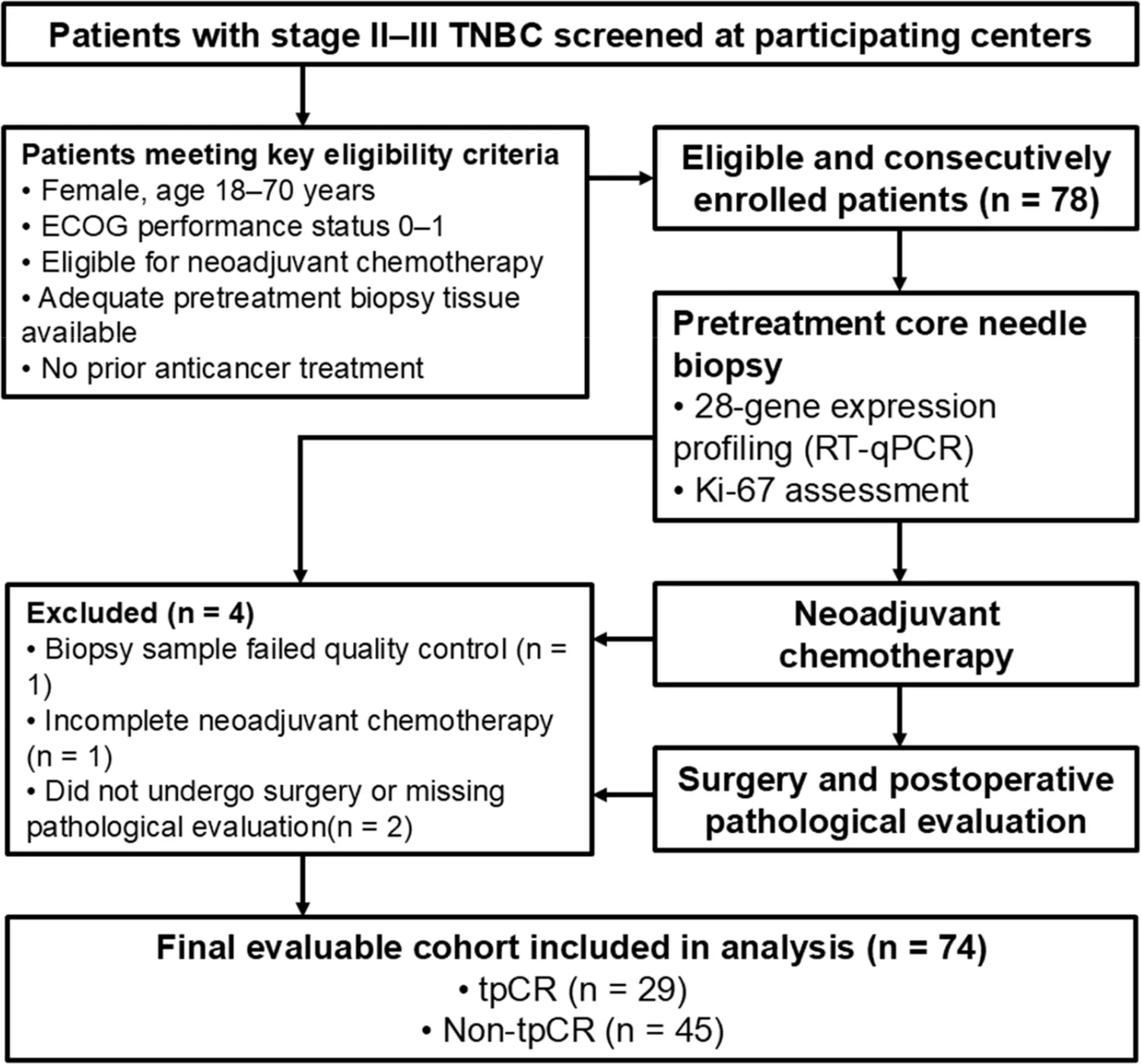
Flow diagram of patient enrollment and study procedures. Patients with stage II–III TNBC were screened and consecutively enrolled at the participating centers. After pretreatment biopsy, neoadjuvant chemotherapy, surgery, and pathological evaluation, four patients were excluded, leaving 74 patients for the final analysis, including 29 with tpCR and 45 with non-tpCR.

### Inclusion and exclusion criteria

Eligible patients were women aged 18–70  with an ECOG performance status of 0–1 and histologically confirmed stage II–III TNBC, as defined in Section 2.3. Additional requirements included availability of adequate pretreatment core-biopsy tissue for molecular testing and Ki-67 assessment, no prior anticancer treatment for the current breast cancer, adequate organ function to receive the planned neoadjuvant chemotherapy, and written informed consent.

Patients were excluded if they had metastatic or inflammatory breast cancer, another malignancy within the previous 5 y (except adequately treated cervical carcinoma in situ or non-melanoma skin cancer), pregnancy or lactation, grade ≥2 peripheral neuropathy, or clinically significant comorbidities or other contraindications precluding the planned neoadjuvant chemotherapy.

### Pathological assessment and biomarker definitions

ER, PR, HER2, and Ki-67 were assessed in pretreatment core-biopsy specimens by the pathology departments of the participating centers using standardized pathological assessment and reporting criteria. ER and PR status was determined according to the percentage of positively stained invasive tumor cell nuclei. ER and PR negativity were defined as <1% nuclear staining, in accordance with the ASCO/CAP recommendations.^16^ HER2-negative status was defined as an IHC score of 0 or 1+, or an IHC score of 2+ without HER2 gene amplification by FISH, according to the ASCO/CAP HER2 testing criteria.^17^ Ki-67 was assessed by immunohistochemistry according to standardized pathological assessment procedures used in the participating centers and Chinese national breast cancer pathology recommendations.^18^ Briefly, the entire slide was first reviewed at low magnification. For tumors with relatively homogeneous staining, at least three high-power fields of invasive carcinoma were evaluated; for tumors with heterogeneous staining, representative fields including areas of higher proliferative activity were assessed. At least 500 invasive tumor cells were counted when applicable. The Ki-67 labeling index was reported as the percentage of invasive tumor cell nuclei showing positive staining.

### Treatment and surgical procedures

All patients received dose-dense anthracycline- and taxane-based NAC. The intensive sequential regimen consisted of epirubicin plus cyclophosphamide (EC) followed by a taxane (paclitaxel or docetaxel), administered at shortened intervals according to the protocol, with supportive care such as granulocyte colony-stimulating factor provided when clinically indicated. Platinum-containing regimens were not routinely used in this cohort. Treatment regimens were adjusted according to patient condition and tolerability, reflecting real-world clinical practice. Surgery was performed within 4 weeks after completion of NAC, including breast-conserving surgery or mastectomy, along with sentinel lymph node biopsy or axillary lymph node dissection. Resected breast and lymph node specimens were subjected to pathological examination to evaluate treatment response. Postoperative adjuvant therapy (including chemotherapy, radiotherapy, or other treatments) was administered according to clinical guidelines (CSCO and Chinese Anti-Cancer Association) and based on baseline staging and pathological response.

### Primary endpoint and pathological response definition

The primary endpoint was tpCR, defined as the absence of residual invasive carcinoma in both the breast and axillary lymph nodes after NAC (ypT0/is, ypN0). Residual ductal carcinoma in situ (DCIS) in the breast was permitted within the definition of tpCR.

### RecurIndex and gene expression analysis

Gene expression analysis was performed using pretreatment core needle biopsy specimens. The RecurIndex assay is a 28-gene RT-qPCR–based expression panel comprising 18 functional target genes and 10 reference genes and was performed using the PanelStation platform. Expression levels of the target genes were normalized against the reference genes according to the predefined assay procedure. The resulting gene-expression profiles were subsequently processed using the established RecurIndex scoring procedure to generate the LRR and DR scores. All samples were processed according to standardized assay procedures and predefined quality-control requirements, and only samples meeting the quality-control criteria were included in the molecular analysis. The RecurIndex assay was provided by Simcere (Jiangsu, China); the company had no role in study design, patient enrollment, data analysis, interpretation of the results, or manuscript preparation.

### Single-cell RNA-seq analysis

Single-cell RNA-seq data from pretreatment TNBC tumor samples were obtained from GSE263995 and analyzed using Seurat. After quality control, doublet removal, normalization, Harmony integration, clustering, and major cell type annotation, the expression distributions of OBSL1, BLM, and CCR1 were evaluated using FeaturePlot and DotPlot analyses. OBSL1-high epithelial tumor-like cells and CCR1-high monocyte/macrophage-lineage cells were defined as cells with expression above the median level among positive cells, whereas cells without detectable expression were defined as low-expression cells. Differential expression analysis was performed between high- and low-expression groups using the Wilcoxon rank-sum test, followed by GO Biological Process, KEGG, and Hallmark enrichment analyses. Because BLM expression was distributed across multiple cell populations, Hallmark module scores related to DNA repair, E2F targets, G2/M checkpoint, mitotic spindle, and MYC targets were calculated using AddModuleScore and compared between BLM-low and BLM-high cells. Cell–cell communication involving CCR1-high myeloid cells was inferred using CellChat based on secreted ligand–receptor interactions.

### Statistical analysis

Associations between assay-generated RecurIndex LRR and DR scores and tpCR were evaluated using univariate logistic regression. For regression analyses, LRR and DR scores were standardized using the Z-score transformation, $Z=\left(X-\overset{®}{X}\right)/\mathrm{SD}$ ; ORs and 95% CIs were reported per 1-SD increase. Raw assay-generated scores were used for distributional comparisons and ROC analyses. Score distributions between the tpCR and non-tpCR groups were summarized as median (interquartile range [IQR]) and compared using the Wilcoxon rank-sum test. Discrimination was assessed using receiver operating characteristic (ROC) curves, with area under the curve (AUC) values and 95% CIs calculated for both LRR and DR scores. As an exploratory subgroup analysis, DR discrimination was further evaluated in patients with cN-negative (cN0) and cN-positive (cN1-3) disease.

Baseline clinicopathological characteristics were compared between the tpCR and non-tpCR groups. Continuous variables were summarized as mean ± SD or median (IQR), as appropriate, and compared using the Wilcoxon rank-sum test. Categorical variables were expressed as counts (percentages) and compared using the Pearson χ^2^ test or Fisher's exact test, as appropriate. Differential expression of the 18 RecurIndex core genes between groups was assessed using the Wilcoxon rank-sum test. Raw *p*-values were reported, with Benjamini–Hochberg false discovery rate (FDR) adjustment performed as a sensitivity analysis.

Univariate logistic regression was used to evaluate associations of age, Ki-67, clinical tumor stage (cT), clinical nodal stage (cN), and candidate genes with tpCR. Candidate predictors for multivariable modeling were selected based on univariate associations, biological plausibility, and model parsimony. Given the limited number of outcome events, model complexity was deliberately restricted. Continuous gene-expression variables were standardized using Z-score transformation, and clinical–molecular models were constructed by combining candidate genes with cN. Model discrimination was evaluated using ROC analysis.

To assess the robustness of variable selection and potential overfitting, LASSO-penalized logistic regression was performed as a sensitivity analysis using an expanded candidate set comprising age, Ki-67, cT, cN, OBSL1, BLM, CCR1, and BUB1B. Continuous variables were standardized, whereas cT and cN were entered as categorical variables. The penalty parameter was selected using stratified five-fold cross-validation with the conservative 1-standard-error (1-SE) rule. Selection stability was assessed across 100 repeated stratified five-fold cross-validations by calculating the proportion of repetitions in which each variable retained a non-zero coefficient.

Internal validation of the best-performing integrated model was performed using 1000 bootstrap resamples to estimate the optimism-corrected AUC. Calibration of the cN-only, OBSL1 + BLM gene-only, and OBSL1 + BLM + cN integrated models was assessed using bootstrap bias-corrected calibration curves based on 1000 resamples, and overall prediction accuracy was quantified using the Brier score. Decision curve analysis (DCA) was performed to explore the potential clinical utility of the integrated model over threshold probabilities ranging from 0.10 to 0.70, with net benefit compared with the cN-only model and the treat-all and treat-none strategies. All statistical tests were two-sided, with *p* < 0.05 considered statistically significant. Analyses were primarily performed using R version 4.5.2, with selected analyses verified using SPSS version 16.0 (SPSS Inc., Chicago, IL, USA).

### Ethics approval and consent to participate

This study was registered with the Chinese Clinical Trial Registry (ChiCTR2000034890). The study was conducted in accordance with the Declaration of Helsinki and approved by the Institutional Review Board (Ethics Committee) of the First Hospital of China Medical University (Ethics Code: AF-SOP-07-1.1-01; approval date: July 13, 2020). Written informed consent was obtained from all patients prior to participation.

## Results

### Baseline patient characteristics and tpCR status

Of the 74 included patients, 29 (39.2%) achieved tpCR. The baseline clinical and pathological characteristics of patients stratified by tpCR status are summarized in Table 1. No statistically significant differences were observed between the tpCR and non-tpCR groups in terms of age at diagnosis, Ki-67 expression level, cT, or histological grade (all *p* > 0.05). The distribution of cN differed significantly between the tpCR and non-tpCR groups according to the overall comparison test shown in Table 1.

**Table 1.** Baseline clinicopathological characteristics of patients stratified by tpCR status.

Variable	Non-pCR *N* = 45	pCR *N* = 29	*p*-value
Age	52.13 ± 10.09	51.93 ± 9.16	0.931
Ki67			1.000
≤30	7 (15.6)	4 (13.8)	
>30	38 (84.4)	25 (86.2)	
**cT**			0.768
1	5 (11.1%)	3 (10.3%)	
2	27 (60.0%)	20 (69%)	
3	12 (26.7%)	6 (21%)	
4	1 (2.2%)	0 (0%)	
**cN**			0.026
0	7 (15.6%)	13 (44.8%)	
1	10 (22.2%)	6 (20.7%)	
2	17 (37.8%)	4 (13.8%)	
3	11 (24.4%)	6 (20.7%)	
**Grade**			0.549
2	18 (40.0%)	9 (31.0%)	
2–3	0 (0.0%)	1 (3.4%)	
3	25 (55.6%)	18 (62.1%)	
Unknown	2 (4.4%)	1 (3.4%)	

### Predictive value of RecurIndex risk scores for tpCR

To evaluate the relationship between the overall RecurIndex risk scores and response to NAC, both the LRR and DR scores were examined before proceeding to individual gene-level analyses. In univariate logistic regression, the DR score was significantly associated with tpCR, with each 1-SD increase corresponding to higher odds of achieving tpCR (OR = 1.668; 95% CI, 1.025–2.851; *p* = 0.048), whereas no significant association was observed for the LRR score (OR = 1.329; 95% CI, 0.828–2.191; *p* = 0.246) (Table 2).

Consistent with these findings, the median DR score was higher in patients who achieved tpCR than in those who did not [33.9 (IQR, 29.3–36.9) vs 28.2 (IQR, 23.9–34.7); *p* = 0.033], whereas the corresponding difference in LRR scores was not statistically significant [40.9 (IQR, 35.2–45.7) vs 38.3 (IQR, 33.6–43.0); *p* = 0.382] (Figure 2A and B). ROC analysis showed modest discrimination of the DR score for tpCR (AUC = 0.648; 95% CI, 0.517–0.780), whereas the LRR score showed limited discrimination (AUC = 0.561; 95% CI, 0.423–0.699) (Figure 2C). In an exploratory analysis stratified by clinical nodal status, DR discrimination was greater in cN-negative patients (*n* = 20; AUC = 0.824; 95% CI, 0.624–1.000) than in cN-positive patients (*n* = 54; AUC = 0.546; 95% CI, 0.365–0.727) (Figure 2D). Although this finding should be interpreted cautiously given the small cN-negative subgroup.

**Table 2.** Univariate logistic regression analysis of RecurIndex risk scores for tpCR.

Predictor	OR^1^	95%CI^2^	*p*-value	*N*
LRR	1.042	0.973, 1.121	0.246	74
LRR(per 1-SD increase)	1.329	0.828, 2.191	0.246	74
DR	1.072	1.003, 1.153	0.048	74
DR (per 1-SD increase)	1.668	1.025, 2.851	0.048	74

**Figure 2.**
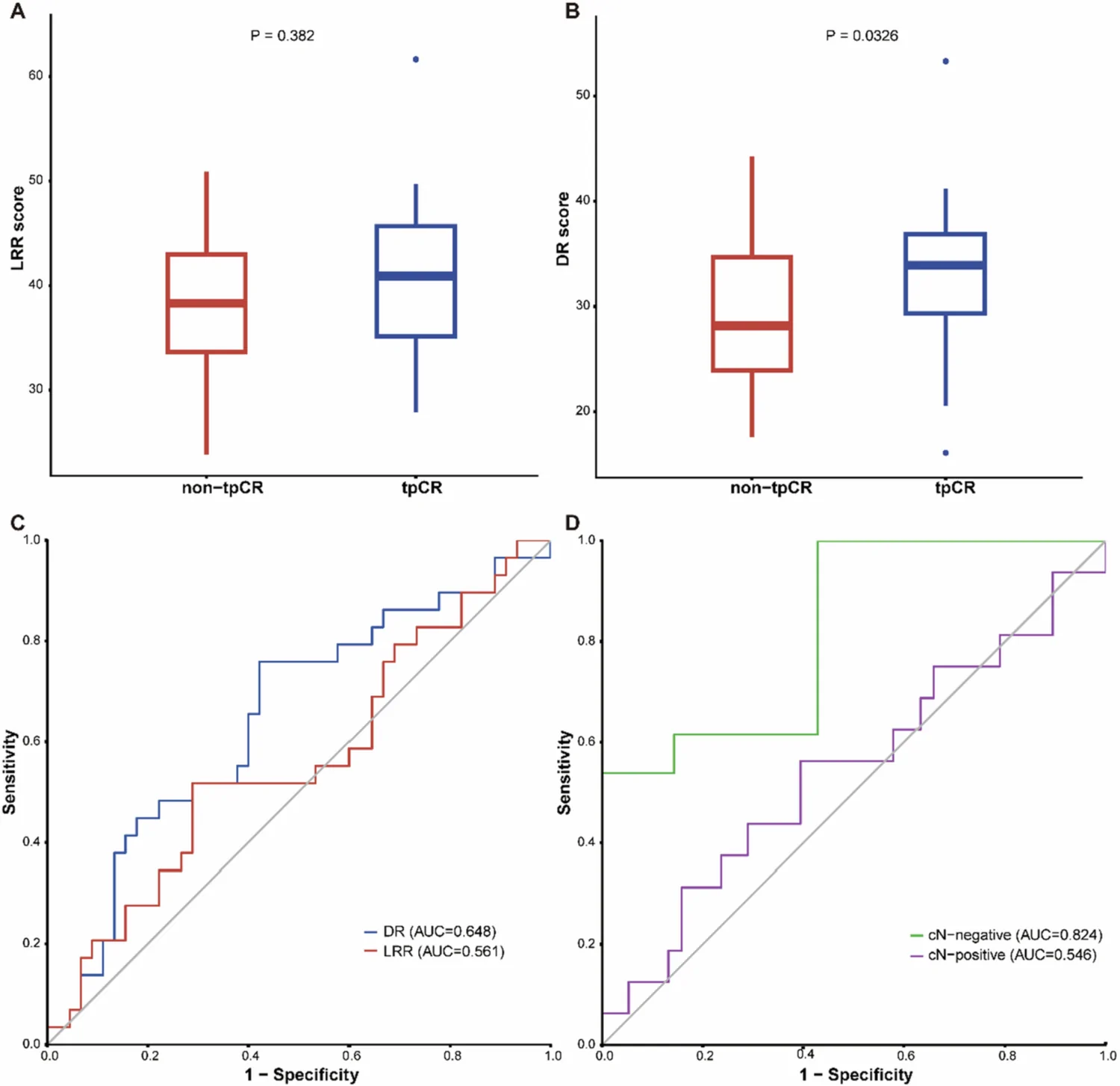
RecurIndex risk scores and prediction of tpCR. (A, B) LRR and DR score distributions according to tpCR status. *p*-values were derived from Wilcoxon rank-sum tests. (C) ROC curves of LRR and DR scores for predicting tpCR. (D) Exploratory ROC analysis of the DR score stratified by clinical nodal status.

### Identification of differentially expressed genes

To identify candidate molecular biomarkers associated with response to NAC, the expression levels of the 18 core genes from the 28-gene panel were compared between patients who achieved tpCR and those who did not. Among the 18 core genes, BLM, OBSL1, CCR1, and BUB1B, showed statistically significant differential expression between the tpCR and non-tpCR groups. Specifically, compared with patients who did not achieve tpCR, those who achieved tpCR exhibited significantly higher expression levels of the four genes (BLM, *p* = 0.003; OBSL1, *p* = 0.007; CCR1, *p* = 0.023; BUB1B, *p* = 0.049) (Figure 3). No statistically significant differences were observed for the remaining genes in the panel. The complete and FDR-adjusted *p*-values for all 18 core genes are provided in Supplementary Table S1. Based on these findings, the four differentially expressed genes were considered candidate biomarkers and were included in subsequent regression analyses to further evaluate their associations with tpCR.

**Figure 3.**
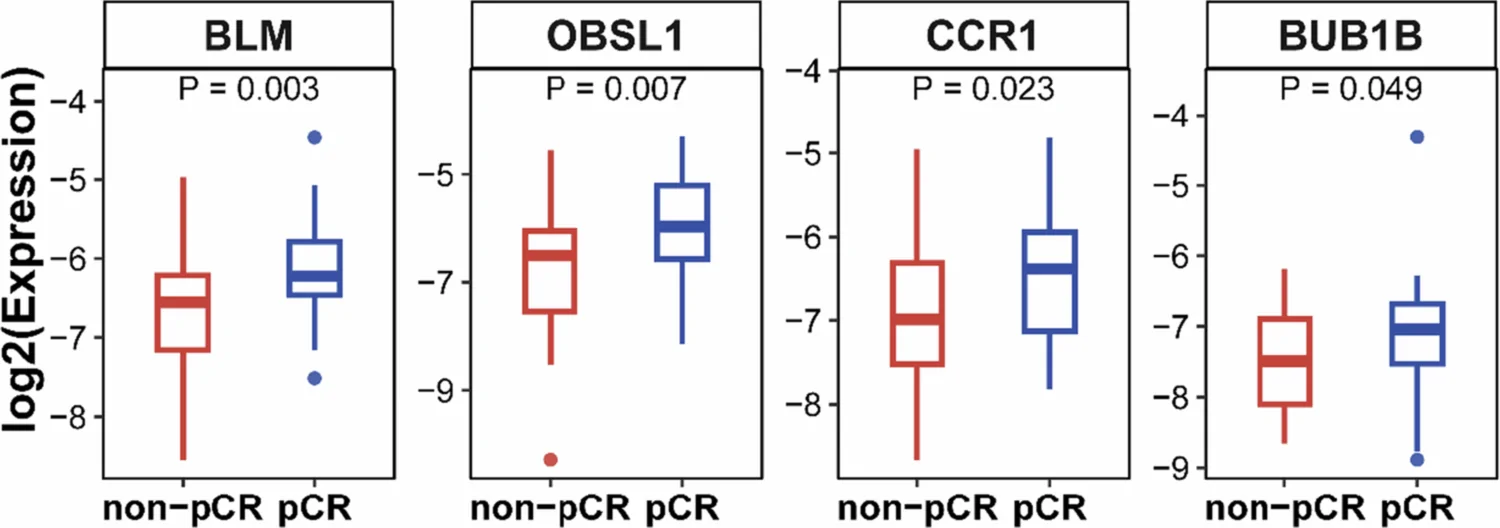
Differential expression of core genes between tpCR and non-tpCR groups. Box plots show log2-transformed expression levels of BLM, OBSL1, CCR1, and BUB1B in patients with or without tpCR. All four genes showed significantly higher expression in the tpCR group than in the non-tpCR group.

### Univariate logistic regression analysis of factors associated with tpCR

To further evaluate factors associated with tpCR following NAC univariate logistic regression analyses were performed for clinicopathological variables and candidate genes identified in Section 3.3 (Table 3). Among clinicopathological factors, age, cT, and Ki-67 index were not significantly associated with tpCR. In contrast, cN stage showed a significant association with tpCR, with patients with higher nodal burden exhibiting a lower likelihood of achieving tpCR. Regarding gene expression variables, higher expression levels of OBSL1, BLM, and CCR1 were significantly associated with tpCR, whereas BUB1B did not reach statistical significance in univariate analysis. Based on these findings, cN and the three genes (OBSL1, BLM, and CCR1) were selected for subsequent multivariable logistic regression analysis.

**Table 3.** Univariate logistic regression analysis of clinicopathological factors and candidate genes associated with tpCR.

Variable	*N*	OR	95%CI	*p*-value
Age	74	1	0.95, 1.05	0.93
**cT**	74			
1	–		–	–
2		1.23	0.27, 6.59	0.789
3		0.83	0.15, 5.18	0.837
**cN**	74			
0	–	–	–	–
1		0.32	0.08, 1.23	0.105
2		0.13	0.03, 0.49	0.004
3		0.29	0.07, 1.10	0.076
Ki67	74	1	0.95, 1.05	0.275
OBSL1	74	2.22	1.31, 4.15	0.006
BLM	74	1.99	1.19, 3.69	0.016
CCR1	74	1.8	1.10, 3.22	0.030
BUB1B	74	2.22	1.05, 5.90	0.092

### Multivariable logistic regression analysis

Based on the univariate analysis results, OBSL1, BLM, and CCR1 were included in a multivariable logistic regression model to evaluate their independent associations with tpCR (Table 4). In the multivariable analysis, OBSL1 remained significantly associated with tpCR, whereas BLM and CCR1 did not reach statistical significance after adjustment. Notably, BLM showed a borderline association with tpCR, while CCR1 demonstrated a weaker and non-significant effect. Despite the attenuation of statistical significance for some genes, all three variables showed a consistent direction of association observed in the univariate analysis. These findings suggest that OBSL1 may serve as an independent predictive factor, while BLM and CCR1 may contribute to tpCR prediction in a combined model.

**Table 4.** Multivariable logistic regression analysis of the three candidate genes associated with tpCR.

Variable	*N*	OR	95%CI	*p*-value
OBSL1	74	1.85	1.03, 3.54	0.047
BLM	74	1.76	0.99, 3.31	0.063
CCR1	74	1.48	0.84, 2.77	0.187

### Construction and validation of the integrated models incorporating clinical factors

Given that cN stage was significantly associated with tpCR in the univariate analysis, it was integrated with candidate genes to construct clinical–molecular models for predicting tpCR. Detailed results of the multivariable logistic regression analyses for the four candidate models are provided in Supplementary Table S2. Among the evaluated models, the OBSL1 + BLM + cN model showed the highest discrimination, with an AUC of 0.838 (Figure 4A). Internal validation using 1000 bootstrap resamples yielded an optimism-corrected AUC of 0.799, indicating that discrimination remained acceptable after correction for optimism.

Calibration was further assessed for the cN-only, OBSL1 + BLM gene-only, and OBSL1 + BLM + cN integrated models using bootstrap bias-corrected calibration curves (Figure 4B and Supplementary Figure S3). The integrated model showed reasonable agreement between predicted and observed probabilities and had the lowest Brier score among the three models (0.156 vs 0.208 and 0.200, respectively). Decision curve analysis showed that the OBSL1 + BLM + cN model provided greater net benefit than the cN-only model and the treat-all and treat-none strategies across threshold probabilities of approximately 0.13–0.70 (Figure 4C).

As a sensitivity analysis for variable selection and potential overfitting, LASSO-penalized logistic regression was performed using an expanded set of clinical and molecular variables. Under the conservative 1-SE criterion, OBSL1, BLM, CCR1, and nodal-stage information retained non-zero coefficients. Across 100 repeated stratified five-fold cross-validations, OBSL1, BLM, and cN showed the highest selection frequencies (79%, 75%, and 74%, respectively), followed by CCR1 (59%), whereas Ki-67 (8%), cT (7%), age (1%), and BUB1B (0%) were infrequently or never retained. The LASSO-penalized regression sensitivity analysis, including penalty parameter selection and variable selection stability, is presented in Supplementary Figure S2. These findings provided additional support for the relative stability of OBSL1, BLM, and cN within the exploratory modeling framework. Overall, integration of RecurIndex-derived molecular features with clinical nodal status showed promising discrimination and potential decision-support value for tpCR prediction, although external validation is required before clinical application.

**Figure 4.**
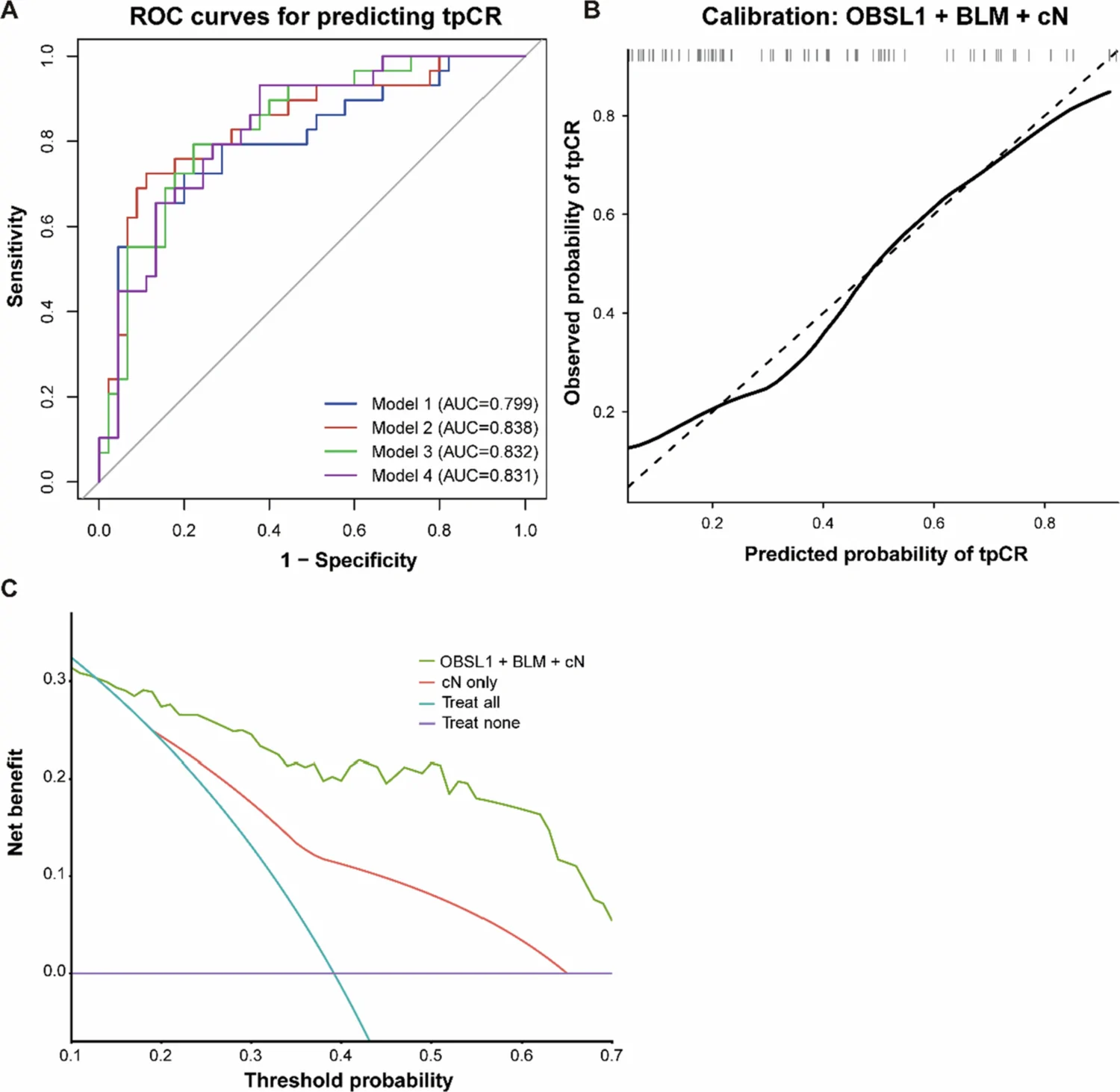
Discrimination, calibration, and clinical utility of integrated gene–clinical models for predicting tpCR in TNBC patients receiving NAC. (A) ROC curves comparing the discriminative performance of different integrated models. (B) Calibration curve of the best-performing model (OBSL1 + BLM +cN), showing agreement between predicted and observed probabilities of tpCR. (C) DCA comparing the net benefit of the OBSL1 + BLM + cN model with the cN-only model and the treat-all and treat-none strategies across threshold probabilities of 0.10–0.70. The integrated model showed greater net benefit than the comparator strategies across thresholds of approximately 0.13–0.70.

### Single-cell transcriptomic characterization of OBSL1, BLM, and CCR1-associated cellular states

Single-cell RNA-seq analysis of pretreatment TNBC samples from GSE263995 identified multiple tumor microenvironment compartments, including epithelial tumor-like cells, monocyte/macrophage-lineage cells, T-cell subsets, B cells, plasma cells, endothelial cells, and fibroblast/pericyte stromal cells (Figure 5A). OBSL1, BLM, and CCR1 showed distinct cellular distribution patterns: OBSL1 was mainly enriched in epithelial tumor-like cells, CCR1 was predominantly localized to monocyte/macrophage-lineage cells, whereas BLM showed broader expression across multiple compartments, with relatively prominent expression in cycling T cells (Figure 5B and C).

**Figure 5.**
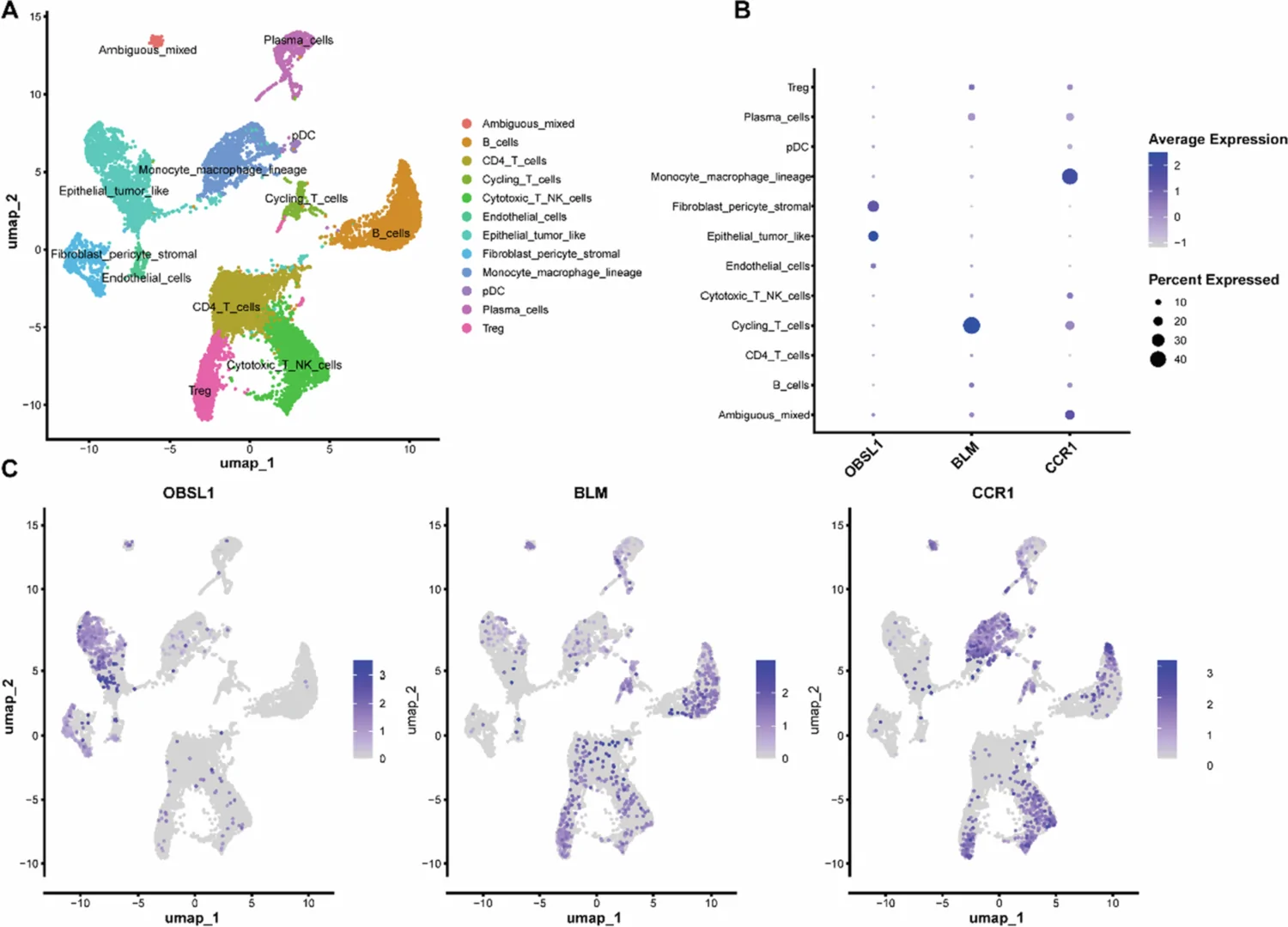
Single-cell characterization of OBSL1, BLM, and CCR1 expression in pretreatment TNBC samples. (A) UMAP visualization of major cell types in pretreatment TNBC tumor samples. (B) Dot plot summarizing the average expression and proportion of cells expressing OBSL1, BLM, and CCR1 across annotated cell types. (C) Expression distribution of OBSL1, BLM, and CCR1 across all cells.

Given that OBSL1 remained independently associated with tpCR in the multivariable analysis, its tumor-cell-intrinsic context was further examined in epithelial tumor-like cells. Compared with OBSL1-low cells, OBSL1-high epithelial tumor-like cells showed enrichment of pathways related to RNA processing, small GTPase signaling, nucleocytoplasmic transport, DNA metabolism, focal adhesion, adherens junctions, and integrin signaling (Figure 6A and B). These findings suggest that OBSL1-high epithelial tumor-like cells represent a transcriptionally active state characterized by adhesion, cytoskeletal signaling, nuclear trafficking, and DNA metabolism-related programs.

Because BLM expression was distributed across multiple cellular compartments, predefined Hallmark module scores were used to evaluate BLM-associated transcriptional programs. BLM-high cells showed higher DNA repair, E2F targets, G2/M checkpoint, mitotic spindle, and MYC targets V1 scores than BLM-low cells (Figure 6C). These programs were observed across multiple cell types, with relatively prominent signatures in cycling T cells and epithelial tumor-like cells (Figure 6D), suggesting that BLM reflects a cross-compartment proliferative and DNA repair-associated transcriptional state.

CCR1 was mainly expressed in monocyte/macrophage-lineage cells; therefore, subsequent analyses focused on CCR1-defined myeloid states. CCR1-high myeloid cells showed enrichment of interferon response, inflammatory and complement-related signaling, IL6/JAK/STAT3 signaling, lysosomal and phagocytic pathways, and efferocytosis (Figure 6E–G). These results suggest that CCR1-high myeloid cells represent an immune-activated state characterized by inflammatory signaling and enhanced phagocytic and lysosomal activity. CellChat analysis was further performed to characterize the communication landscape of CCR1-high myeloid cells. Predicted interactions were observed with multiple immune, epithelial, endothelial, and stromal compartments, with relatively prominent communication involving cytotoxic T/NK cells, epithelial tumor-like cells, and stromal cells (Figure 6H). These findings suggest that CCR1-high myeloid cells are embedded in an active tumor–immune–stromal communication network within pretreatment TNBC tumors. Additional differential expression and pathway enrichment analyses of OBSL1-high epithelial tumor-like cells and CCR1-high myeloid cells are shown in Supplementary Figure S1.

**Figure 6.**
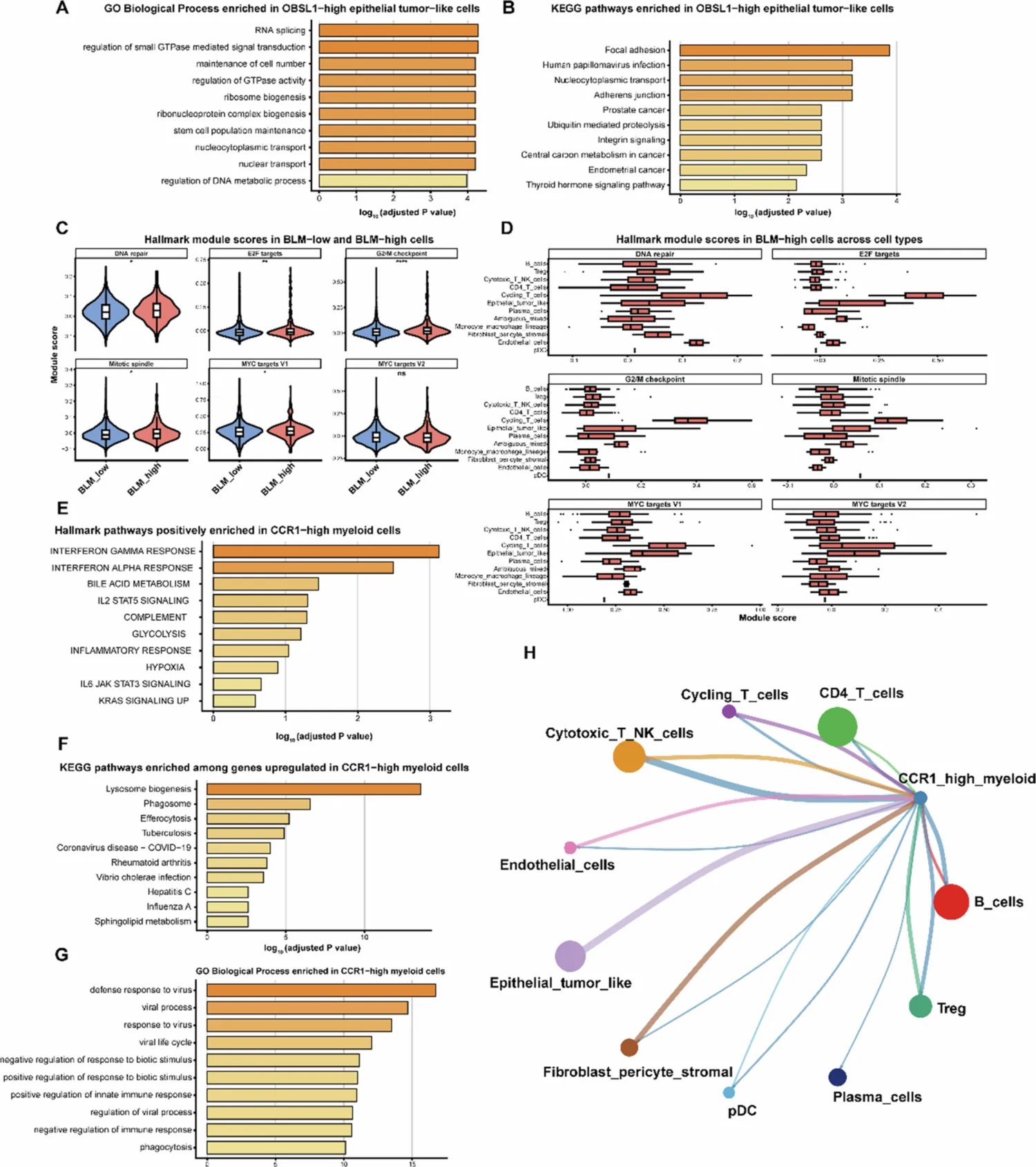
Single-cell characterization of OBSL1, BLM and CCR1-associated cellular states in pretreatment TNBC samples. (A, B) GO Biological Process and KEGG pathway enrichment analyses of genes upregulated in OBSL1-high epithelial tumor-like cells. (C) Hallmark module scores comparing BLM-low and BLM-high cells. (D) Hallmark module scores among BLM-high cells across major cell types. (E–G) Hallmark, KEGG, and GO biological process enrichment analyses of genes upregulated in CCR1-high monocyte/macrophage-lineage cells. (H) CellChat-inferred communication network centered on CCR1-high myeloid cells.

## Discussion

RecurIndex, a multigene assay developed using tumor samples from Asian populations, has primarily been applied to postoperative recurrence-risk assessment and adjuvant treatment decision-making in hormone receptor-positive, HER2-negative breast cancer.^9^ Its utility in the neoadjuvant setting of TNBC remains insufficiently characterized. In the present study, the DR score was significantly associated with tpCR, and DR showed a higher observed AUC for treatment response. These findings suggest that the two RecurIndex outputs may capture partly distinct biological dimensions, with molecular features related to distant recurrence potentially overlapping more closely with processes relevant to systemic chemotherapy responsiveness. Exploratory stratification further showed a higher DR AUC in cN-negative than in cN-positive patients. Given the small cN-negative subgroup, however, this observation should be considered hypothesis-generating and requires independent validation.

At the individual-gene level, OBSL1, BLM, and CCR1 emerged as the principal candidates associated with tpCR. BLM is involved in DNA damage repair and genomic stability,^19^ ^,^ ^20^ whereas CCR1 participates in immune and myeloid-cell signaling within the tumor microenvironment.^21^ OBSL1 is a cytoskeletal adaptor implicated in microtubule dynamics and genome integrity, suggesting a potential role in cellular processes relevant to tumor behavior.^22^ ^,^ ^23^ In multivariable analysis, OBSL1 remained independently associated with tpCR, while the effects of BLM and CCR1 were attenuated after adjustment, suggesting that treatment response is unlikely to be adequately represented by a single molecular marker.

Single-cell transcriptomic analysis provided additional biological context for these candidates. OBSL1 was predominantly expressed in epithelial tumor-like cells and was associated with adhesion, cytoskeletal, nucleocytoplasmic transport, and DNA metabolism-related programs. BLM was distributed across multiple cellular compartments and was linked to proliferative and DNA repair-associated states, whereas CCR1 was mainly localized to monocyte/macrophage-lineage cells characterized by inflammatory, interferon, phagocytic, and lysosomal programs. Together, these findings suggest that the candidate genes may reflect complementary tumor-intrinsic and immune-associated states relevant to chemotherapy response. These analyses remain exploratory because they were derived from a limited number of publicly available pretreatment samples and computational pathway inference.

Integration of cN stage with molecular features improved model discrimination, with the OBSL1 + BLM + cN model showing the highest performance among the evaluated models. Discrimination remained acceptable after bootstrap correction for optimism, and the integrated model showed reasonable calibration and the lowest Brier score compared with the cN-only and gene-only models. Repeated LASSO analyses further showed that OBSL1, BLM, and cN were among the most consistently retained predictors, providing complementary evidence for their relative stability within the exploratory modeling framework. DCA further suggested incremental decision-support value over cN alone. Conceptually, patients with a higher predicted probability of tpCR may be adequately managed with standard NAC, whereas those with a lower predicted probability may warrant evaluation of intensified or alternative strategies in future studies. Because no validated tpCR probability threshold currently guides treatment selection in TNBC, these findings remain exploratory and do not establish a clinical treatment cutoff.

Several limitations should be acknowledged. First, despite the multicenter design, the sample size and number of tpCR events were limited, and the prediction model lacks independent external validation. Second, the molecular findings were based on transcriptomic measurements without protein-level or functional validation. Third, the substantial molecular heterogeneity of TNBC, including immune-activated, basal-like, and androgen receptor-positive subtypes,^24^ may not be fully captured by the current clinical-molecular model. Larger independent TNBC cohorts incorporating complementary molecular data are therefore needed to validate and further refine these findings.

In conclusion, this exploratory multicenter study suggests that the RecurIndex-derived DR score, together with selected molecular features including OBSL1 and BLM, is associated with tpCR after dose-dense or dose-intensified NAC in TNBC. Integration of molecular features with clinical nodal status showed promising discrimination for tpCR prediction, while single-cell analyses provided complementary biological context for the identified candidates. These findings are hypothesis-generating and require validation in larger independent TNBC-specific cohorts.

## Supplementary Material

Supplementary material STROBE checklist_v4_cohort.doc

Supplementary material Supplementary figure S1.docx

Supplementary material Supplementary Tables S2.docx

Supplementary material Supplementary figure S2.docx

Supplementary material Supplementary Table S1.docx

Supplementary figure S3.docx Supplementary figure S3.docx

## Data Availability

All data supporting the findings of this study are available from the corresponding author upon reasonable request, subject to institutional review board approval. scRNA-Seq data were deposited into the Gene Expression Omnibus database under accession number GSE263995 and are available at the following URL: https://www.ncbi.nlm.nih.gov/geo/query/acc.cgi?acc=GSE263995
