# Supplementary material for: RecurIndex-derived molecular features associated with total pathological complete response in triple-negative breast cancer: An exploratory prospective cohort study with single-cell transcriptomic contextualization
Source: Cancer Biol Ther. 2026 Oct 10;27(1):2743407. doi: 10.1080/15384047.2026.2743407 (PMC13656812; doi:10.1080/15384047.2026.2743407)
Supplement: Supplementary file 1 [file KCBT_A_2743407_SM5115.docx]

**Figure S1. Supporting single-cell analyses for OBSL1 and CCR1 high-expression states**

(A) Volcano plot of OBSL1-high versus OBSL1-low epithelial tumor-like cells
(B) Hallmark enrichment analysis of OBSL1-high epithelial tumor-like cells
(C) Volcano plot of CCR1-high versus CCR1-low myeloid cells
