# Supplementary material for: RecurIndex-derived molecular features associated with total pathological complete response in triple-negative breast cancer: An exploratory prospective cohort study with single-cell transcriptomic contextualization
Source: Cancer Biol Ther. 2026 Oct 10;27(1):2743407. doi: 10.1080/15384047.2026.2743407 (PMC13656812; doi:10.1080/15384047.2026.2743407)
Supplement: Supplementary file 2 [file KCBT_A_2743407_SM5116.docx]

**Figure S2. LASSO-penalized regression sensitivity analysis for tpCR prediction.**

(A) Five-fold cross-validation for selection of the LASSO penalty parameter.

(B) Selection frequencies of clinical and molecular variables across 100 repeated stratified five-fold cross-validations using the conservative 1-standard-error (1-SE) criterion.
