# Supplementary material for: RecurIndex-derived molecular features associated with total pathological complete response in triple-negative breast cancer: An exploratory prospective cohort study with single-cell transcriptomic contextualization
Source: Cancer Biol Ther. 2026 Oct 10;27(1):2743407. doi: 10.1080/15384047.2026.2743407 (PMC13656812; doi:10.1080/15384047.2026.2743407)
Supplement: Supplementary file 3 [file KCBT_A_2743407_SM5118.docx]

**Table S2. Multivariate logistic regression analysis of four models with different variable combinations.**

| Model | term | OR | CI | P |
| --- | --- | --- | --- | --- |
| Model 1 | OBSL1 | 2.61 | 1.5, 5.13 | 0.002 |
| Model 1 | cN_1 | 0.29 | 0.06, 1.26 | 0.108 |
| Model 1 | cN_2 | 0.07 | 0.01, 0.34 | 0.002 |
| Model 1 | cN_3 | 0.2 | 0.04, 0.83 | 0.032 |
| Model 2 | OBSL1 | 2.29 | 1.27, 4.63 | 0.01 |
| Model 2 | BLM | 2.12 | 1.09, 4.52 | 0.037 |
| Model 2 | cN_1 | 0.32 | 0.06, 1.45 | 0.147 |
| Model 2 | cN_2 | 0.05 | 0.01, 0.27 | 0.002 |
| Model 2 | cN_3 | 0.24 | 0.05, 1.01 | 0.06 |
| Model 3 | OBSL1 | 2.21 | 1.21, 4.53 | 0.016 |
| Model 3 | CCR1 | 1.9 | 1.01, 4.13 | 0.069 |
| Model 3 | cN_1 | 0.31 | 0.06, 1.32 | 0.123 |
| Model 3 | cN_2 | 0.05 | 0.01, 0.28 | 0.001 |
| Model 3 | cN_3 | 0.16 | 0.03, 0.71 | 0.022 |
| Model 4 | BLM | 2.35 | 1.24, 5.03 | 0.016 |
| Model 4 | CCR1 | 2.25 | 1.22, 4.84 | 0.019 |
| Model 4 | cN_1 | 0.33 | 0.07, 1.46 | 0.153 |
| Model 4 | cN_2 | 0.04 | 0, 0.23 | 0.001 |
| Model 4 | cN_3 | 0.23 | 0.05, 1.01 | 0.059 |
