# Supplementary material for: RecurIndex-derived molecular features associated with total pathological complete response in triple-negative breast cancer: An exploratory prospective cohort study with single-cell transcriptomic contextualization
Source: Cancer Biol Ther. 2026 Oct 10;27(1):2743407. doi: 10.1080/15384047.2026.2743407 (PMC13656812; doi:10.1080/15384047.2026.2743407)
Supplement: Supplementary file 4 [file KCBT_A_2743407_SM5119.docx]

**Table S1. Complete P-values of differential expression analysis for the 18 core genes between pCR and non-pCR groups.**

| Gene | n_non_pCR | n_pCR | p_value | FDR_BH |
| --- | --- | --- | --- | --- |
| BLM | 45 | 29 | 0.00340271 | 0.06124874 |
| OBSL1 | 45 | 29 | 0.00712952 | 0.06416565 |
| CCR1 | 45 | 29 | 0.02321204 | 0.13927222 |
| BUB1B | 45 | 29 | 0.04872337 | 0.21925516 |
| MMP15 | 45 | 29 | 0.09451804 | 0.34026495 |
| NFATC2IP | 45 | 29 | 0.18030379 | 0.47291619 |
| TCF3 | 45 | 29 | 0.18391185 | 0.47291619 |
| DTX2 | 45 | 29 | 0.39386701 | 0.66875453 |
| STIL | 45 | 29 | 0.43176808 | 0.66875453 |
| RCHY1 | 45 | 29 | 0.46828688 | 0.66875453 |
| TRPV | 45 | 29 | 0.47851594 | 0.66875453 |
| TPX2 | 45 | 29 | 0.4854257 | 0.66875453 |
| PIM1 | 45 | 29 | 0.49939208 | 0.66875453 |
| DDX39 | 45 | 29 | 0.55358579 | 0.66875453 |
| C16ORF7 | 45 | 29 | 0.55729544 | 0.66875453 |
| PTI1 | 45 | 29 | 0.88553677 | 0.99558261 |
| ERBB2 | 45 | 29 | 0.9735 | 0.99558261 |
| ESR1 | 45 | 29 | 0.99558261 | 0.99558261 |
