# Supplementary material for: RecurIndex-derived molecular features associated with total pathological complete response in triple-negative breast cancer: An exploratory prospective cohort study with single-cell transcriptomic contextualization
Source: Cancer Biol Ther. 2026 Oct 10;27(1):2743407. doi: 10.1080/15384047.2026.2743407 (PMC13656812; doi:10.1080/15384047.2026.2743407)
Supplement: Supplementary file 5 [file KCBT_A_2743407_SM5120.docx]

**Figure S3. Calibration of clinical, molecular, and integrated models for predicting tpCR.**

(A) cN-only model.

(B) OBSL1 + BLM gene-only model.

(C) OBSL1 + BLM + cN integrated model. Calibration was internally assessed using 1,000 bootstrap resamples; the dashed diagonal line represents ideal calibration.
