# Supplementary material for: RecurIndex-derived molecular features associated with total pathological complete response in triple-negative breast cancer: An exploratory prospective cohort study with single-cell transcriptomic contextualization
Source: Cancer Biol Ther. 2026 Oct 10;27(1):2743407. doi: 10.1080/15384047.2026.2743407 (PMC13656812; doi:10.1080/15384047.2026.2743407)
Supplement: Supplementary file 6 [file KCBT_A_2743407_SM5117.doc]

STROBE Statement—Checklist of items that should be included in reports of ***cohort studies***

|  | Item No | Recommendation | Location in manuscript |
| --- | --- | --- | --- |
| **Title and abstract** | 1 | (*a*) Indicate the study’s design with a commonly used term in the title or the abstract | Title page & Abstract |
|  |  | (*b*) Provide in the abstract an informative and balanced summary of what was done and what was found | Abstract |
| Introduction | | |  |
| Background/rationale | 2 | Explain the scientific background and rationale for the investigation being reported | Section 1.Introduction |
| Objectives | 3 | State specific objectives, including any prespecified hypotheses | Section 1.Introduction |
| Methods | | |  |
| Study design | 4 | Present key elements of study design early in the paper | Section 2.1 Study Design and Study Population |
| Setting | 5 | Describe the setting, locations, and relevant dates, including periods of recruitment, exposure, follow-up, and data collection | Section 2.1 Study Design and Study Population |
| Participants | 6 | (*a*) Give the eligibility criteria, and the sources and methods of selection of participants. Describe methods of follow-up | Section 2.1 Study Design and Study Population; 2.2 Inclusion and Exclusion Criteria |
|  |  | (*b*) For matched studies, give matching criteria and number of exposed and unexposed | No case matching was performed in this cohort |
| Variables | 7 | Clearly define all outcomes, exposures, predictors, potential confounders, and effect modifiers. Give diagnostic criteria, if applicable | Section 2.4 Endpoint Definition + 2.5 RecurIndex Analysis |
| Data sources/ measurement | 8* | For each variable of interest, give sources of data and details of methods of assessment (measurement). Describe comparability of assessment methods if there is more than one group | Section 2.5 RecurIndex and Gene Expression Analysis |
| Bias | 9 | Describe any efforts to address potential sources of bias | Section 2.1: unified research protocol; Uniform pathological tpCR evaluation; Multivariate adjustment for clinical confounders |
| Study size | 10 | Explain how the study size was arrived at | Section 2.1: EPV principle, expected 30 tp events based on published 30%–40% TNBC pCR rate |
| Quantitative variables | 11 | Explain how quantitative variables were handled in the analyses. If applicable, describe which groupings were chosen and why | Statistical Analysis: RecurIndex scores standardized by 1-SD; Gene expression normalized via Z-score transformation |
| Statistical methods | 12 | (*a*) Describe all statistical methods, including those used to control for confounding | Section 2.6 Statistical Analysis: Baseline comparisons used Wilcoxon rank-sum test (continuous) and χ²/Fisher’s exact test (categorical). Univariate and multivariable binary logistic regression were adopted for binary tpCR outcome; clinical nodal stage (cN) was included to adjust confounding effects of tumor lymphatic burden. RecurIndex risk scores and gene expression values were Z-score standardized prior to regression. ROC-AUC, bootstrap calibration curves and DCA were applied to evaluate model performance; Benjamini–Hochberg FDR correction was used for multiple gene comparisons. All analyses were conducted in R v4.5.2 and partially verified with SPSS 16.0. |
|  |  | (*b*) Describe any methods used to examine subgroups and interactions | No clinical subgroup stratification or interaction tests between clinical/molecular predictors were conducted in logistic regression models. |
|  |  | (*c*) Explain how missing data were addressed | Follow strict sample QC filtering; Only fully qualified biopsy samples included, no missing gene expression data, |
|  |  | (*d*) If applicable, explain how loss to follow-up was addressed | All enrolled patients completed NAC and definitive surgery, no loss to follow-up |
|  |  | (*e*) Describe any sensitivity analyses | Section 2.6: FDR correction for differential gene expression; Bootstrap internal validation (1000 resamples) for predictive models |
| Results | | |  |
| Participants | 13* | (a) Report numbers of individuals at each stage of study—eg numbers potentially eligible, examined for eligibility, confirmed eligible, included in the study, completing follow-up, and analysed | Total screened 78, excluded 4, final analytical cohort n=74; tpCR=29, non-tpCR=45 |
|  |  | (b) Give reasons for non-participation at each stage | Figure 1: Exclusion causes: incomplete NAC, biopsy QC failure, no surgical resection |
|  |  | (c) Consider use of a flow diagram | Figure 1: Flowchart of patient enrollment and exclusion workflow |
| Descriptive data | 14* | (a) Give characteristics of study participants (eg demographic, clinical, social) and information on exposures and potential confounders | Table 1 Baseline characteristics |
|  |  | (b) Indicate number of participants with missing data for each variable of interest | All clinical and RecurIndex gene expression data were complete without missing cases |
|  |  | (c) Summarise follow-up time (eg, average and total amount) | Section 2.3: All patients received surgery within 4 weeks post-NAC; Uniform pathological assessment timepoint |
| Outcome data | 15* | Report numbers of outcome events or summary measures over time | Section 3.1: 29 out of 74 patients achieved total pathological complete response (tpCR, 39.2%) |
| Main results | 16 | (*a*) Give unadjusted estimates and, if applicable, confounder-adjusted estimates and their precision (eg, 95% confidence interval). Make clear which confounders were adjusted for and why they were included | Table 2, Table 3, Table 4 : OR values with 95%CI from univariate and multivariate logistic regression |
|  |  | (*b*) Report category boundaries when continuous variables were categorized | Table 1:Ki-67 grouped as ≤30 / >30; cT/cN clinical staging categorical grouping per TNM standard |
|  |  | (*c*) If relevant, consider translating estimates of relative risk into absolute risk for a meaningful time period | Study focused on predictive odds ratios for binary tpCR endpoint, absolute risk conversion not performed |
| Other analyses | 17 | Report other analyses done—eg analyses of subgroups and interactions, and sensitivity analyses | Section 3.3 differential gene expression with FDR correction; Section 3.6 model comparison, bootstrap internal validation, calibration and DCA |
| Discussion | | |  |
| Key results | 18 | Summarise key results with reference to study objectives | Section 4: RecurIndex DR score and OBSL1 independent tpCR predictive value, single-cell cellular mechanism |
| Limitations | 19 | Discuss limitations of the study, taking into account sources of potential bias or imprecision. Discuss both direction and magnitude of any potential bias | Section 4: small exploratory sample, public single-cell dataset only, lack protein validation |
| Interpretation | 20 | Give a cautious overall interpretation of results considering objectives, limitations, multiplicity of analyses, results from similar studies, and other relevant evidence | Section 4: Biological function of BLM/OBSL1/CCR1 integrated with published breast cancer research |
| Generalisability | 21 | Discuss the generalisability (external validity) of the study results | Section 4: Results require validation in larger multi-center independent TNBC cohorts |
| Other information | | |  |
| Funding | 22 | Give the source of funding and the role of the funders for the present study and, if applicable, for the original study on which the present article is based | Funding information is reported in the acknowledgments. RecurIndex detection kits were supplied by Simcere (Jiangsu, China). Neither the funding bodies nor the assay manufacturer participated in study design, sample testing, statistical analysis, result interpretation, or manuscript writing |

*Give information separately for exposed and unexposed groups.

**Note:** An Explanation and Elaboration article discusses each checklist item and gives methodological background and published examples of transparent reporting. The STROBE checklist is best used in conjunction with this article (freely available on the Web sites of PLoS Medicine at http://www.plosmedicine.org/, Annals of Internal Medicine at http://www.annals.org/, and Epidemiology at http://www.epidem.com/). Information on the STROBE Initiative is available at http://www.strobe-statement.org.
